# *Agrobacterium*-mediated and electroporation-mediated transformation of *Chlamydomonas reinhardtii:* a comparative study

**DOI:** 10.1186/s12896-018-0416-3

**Published:** 2018-02-17

**Authors:** Paola Mini, Olivia Costantina Demurtas, Silvia Valentini, Patrizia Pallara, Giuseppe Aprea, Paola Ferrante, Giovanni Giuliano

**Affiliations:** 1 ENEA, Italian National Agency for New Technologies, Energy and Sustainable Economic Development, Casaccia Research Center, 00123 Rome, Italy; 2 University of Rome “La Sapienza”, Piazzale Aldo Moro, 5, 00185 Rome, Italy

**Keywords:** *Chlamydomonas*, *Agrobacterium*, Gene expression, Luciferase, Silencing

## Abstract

**Background:**

*Chlamydomonas reinhardtii* is an unicellular green alga used for functional genomics studies and heterologous protein expression. A major hindrance in these studies is the low level and instability of expression of nuclear transgenes, due to their rearrangement and/or silencing over time.

**Results:**

We constructed dedicated vectors for *Agrobacterium*-mediated transformation carrying, within the T-DNA borders, the *Paromomycin* (*Paro*) selectable marker and an expression cassette containing the *Luciferase* (*Luc*) reporter gene. These vectors and newly developed co-cultivation methods were used to compare the efficiency, stability and insertion sites of *Agrobacterium*- versus electroporation-mediated transformation. The influence of different transformation methods, of the cell wall, of the virulence of different *Agrobacterium* strains, and of transgene orientation with respect to T-DNA borders were assessed. False positive transformants were more frequent in *Agrobacterium*-mediated transformation compared to electroporation, compensating for the slightly lower proportion of silenced transformants observed in *Agrobacterium*-mediated transformation than in electroporation. The proportion of silenced transformants remained stable after 20 cycles of subculture in selective medium. Next generation sequencing confirmed the nuclear insertion points, which occurred in exons or untraslated regions (UTRs) for 10 out of 10 *Agrobacterium*-mediated and 9 out of 13 of electroporation-mediated insertions. Electroporation also resulted in higher numbers of insertions at multiple *loci.*

**Conclusions:**

Due to its labor-intensive nature, *Agrobacterium* transformation of *Chlamydomonas* does not present significant advantages over electroporation, with the possible exception of its use in insertional mutagenesis, due to the higher proportion of within-gene, single-locus insertions. Our data indirectly support the hypothesis that rearrangement of transforming DNA occurs in the *Chlamydomonas* cell, rather than in the extracellular space as previously proposed.

**Electronic supplementary material:**

The online version of this article (10.1186/s12896-018-0416-3) contains supplementary material, which is available to authorized users.

## Background

*Chlamydomonas reinhardtii* is widely used for functional genomics studies as well as for heterologous protein production. Significant progress has been made since the first transformation reports using complementation of the *nitrate reductase* (*nit1*) and *argininosuccinate lyase* (*arg7*) mutations [1, 2]. A large number of selectable markers and promoters have been used for nuclear transformation, and delivery of foreign DNA has been obtained through a variety of methods, such as particle bombardment [1], agitation with glass beads or silicon carbide whiskers [3, 4], electroporation [5], or *Agrobacterium tumefaciens* [6–8]. Despite recent advances in genetic transformation, the expression of foreign genes in the nuclear genome of wild-type *Chlamydomonas* remains challenging due to transgene rearrangement and silencing [8–10].

In this scenario, the possibility to transform *Chlamydomonas* by *Agrobacterium tumefaciens* is appealing, given the lower levels of transgene rearrangement and silencing reported in plants. *Agrobacterium tumefaciens* is a pathogenic soil bacterium that has evolved the capacity to transfer a segment of DNA (the T-DNA) from the tumor-inducing (Ti) plasmid into the nucleus of a plant cell. An advantage in using *Agrobacterium* for plant transformation is the reduction in transgene copy number, DNA rearrangements and transgene silencing [11, 12].

Nuclear transformation of *Chlamydomonas reinhardtii* by *Agrobacterium tumefaciens* has been reported, using vectors containing the Cauliflower Mosaic Virus *35S* promoter and non-codon-optimized reporter genes [6]. The reported transformation efficiency was 50-fold higher than that obtained with the glass beads method. Further improvements of the transformation efficiency were described in a second report [7] using modified transformation vectors, culture media and *Agrobacterium* strains. No data on the stability of expression over time of the reporter gene were reported in either case.

In the present work, we describe the construction of transformation vectors carrying a selectable marker (*Paro* gene [13]) and a reporter gene (*Renilla reniformis* luciferase (*Luc*) [14]) which are codon optimized for *Chlamydomonas* nuclear expression and placed under the control of strong *Chlamydomonas* promoters: *70 kDa heat shock protein/ribulose bisphosphate carboxylase/oxygenase small subunit* (*HSP70/RBCS2)* [15] and *Photosystem I reaction center subunit II* (*PSAD*) [16]. The effects of different transformation protocols, *Agrobacterium* strains, and orientation of the transgenes with respect to the T-DNA right border were studied, and compared with results obtained via electroporation.

## Results

### Development of vectors and protocols for *Agrobacterium*-mediated transformation of *C. reinhardtii*

**Fig. 1:**
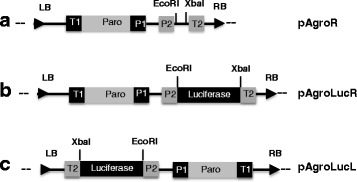
Schematic map of the vectors used for *Agrobacterium*-mediated transformation. Only the portion between the LB and RB (T-DNA) is shown. All vectors contain the *Paro* gene, conferring resistance to paromomycin under the control of the strong *HSP70/RBCS2* hybrid promoter (P1) and *RBCS2* terminator (T1). pAgroR (**a**) contains an expression cassette containing the *PSAD* promoter (P2) and terminator (T2) sequences. In pAgroLucR (**b**) the *Luc* coding sequence is cloned in the expression cassette. In pAgroLucL (**c**) the *Paro* and *Luc* cassettes have an inverted orientation with respect to pAgroLucR. For further details see Material and Methods

First, we constructed the pAgroR vector, optimized for *Agrobacterium*-mediated *Chlamydomonas* transformation. This vector harbors the *Paro* gene, conferring paromomycin resistance [13] and an empty expression cassette consisting of the *PSAD* promoter and terminator [16] flanking a multiple cloning site (Fig. 1a). The vector was introduced in two *Agrobacterium* strains with low and high virulence, respectively, LBA4404 and C58C1 [17] and used for transformation of the cell wall-proficient CC125(+) and the cell wall-deficient cw15 *Chlamydomonas* strains.

In the first round of experiments we followed published co-cultivation protocols [6, 7], but we were unable to obtain paromomycin-resistant colonies. Since *Agrobacterium virulence (vir)* genes are known to be induced by acidic pH and nutrient-poor growth media [18], we developed a different transformation method, based on the recommendations by Gelvin [19]. This method consists in growing *Agrobacterium* cells for the first 24 h in AB medium and then overnight in AB induction medium [19]. Co-cultivation of *Agrobacterium* with *Chlamydomonas* is carried out on solid AB induction medium for 48 h (for further details see Materials and Methods).

Using this method, cw15 cells were successfully transformed using both the LBA4404 and C58C1 *Agrobacterium* strains, while CC125(+) cells could be transformed only with the more virulent C58C1 strain (Table 1). In general, higher transformation efficiencies were obtained using cell wall-deficient *Chlamydomonas* cells and highly virulent *Agrobacterium* (Table 1). Transformation was confirmed by PCR analysis using oligonucleotides directed to the *Paro* gene (See Additional file 1: Figure S1 for an example). Sixty-seven to 92 % (67–92%) of paromomycin-resistant colonies were PCR-positive for the *Paro* gene (Table 1).

**Table 1:** Efficiency of different *Agrobacterium*-mediated transformation methods. Two different *Agrobacterium* strains (LBA4404 and C58C1), containing the pAgroR vector and two *Chlamydomonas* strains (CC125 and cw15) were used. Transformation efficiency is expressed as the number of colonies resistant to paromomycin/10^8^ cells transformed. The presence of the *Paro* transgene was tested by PCR and is expressed as the percentage of *Paro*-resistant colonies testing positive in the PCR assay. *Kanamycin (Kan)*-positive transformants, indicative of bacterial contamination, were excluded from the analysis. Results of two independent experiments for each protocol/strain combination are reported. At least 50 independent transformants for each experiment were analyzed by PCR

*Chlamydomonas* strain	*Agrobacterium* strain	Transformation efficiency (colonies/10^8^ cells)	Positivity to *Paro* (PCR)
cw15	LBA4404	31	75%
		16	92%
cw15	C58C1	25	75%
		33	67%
CC125	LBA4404	1	0%
		0	/
CC125	C58C1	6	86%
		14	92%

We conducted a second round of experiments using the pAgroLucR vector, carrying a codon-optimized luciferase (*Luc*) gene [14] in the *PSAD* expression cassette (Fig. 1b). This vector was used for either *Agrobacterium*-mediated transformation or electroporation of the cw15 strain (Table 2). Even with the optimized protocol, *Agrobacterium*-mediated transformation efficiencies were 2.5- to 60-fold lower with respect to electroporation using the same plasmid (Table 2). Also, while 100% of electroporated Paro-resistant colonies tested are positive for the presence of the *Paro* gene in the PCR assay, only 65% to 97% of colonies obtained after co-cultivation with *Agrobacterium* did so, indicating a high percentage of false positive transformants in the latter protocol (Table 2).

**Table 2:** Comparison of electroporation and *Agrobacterium*-mediated transformation*.* Transformation efficiencies are expressed as the number of paromomycin resistant colonies/10^8^ cells transformed. Presence of *Paro* and *Luc* transgenes was tested by PCR. Transformants containing the *Luc* transgene were further analyzed for Luc enzymatic activity (last column). Results of two independent experiments are reported. At least 96 independent transformants were analyzed for each experiment

Transformation method	Plasmid	Transformants/10^8^ cells	*Paro*-positive transformants (PCR)	*Luc-*positive transformants (PCR)	Transformants exhibiting Luc activity^a^
Electroporation	pAgroLucR	120	100%	26%	15%
		250	100%	33%	20%
*Agrobacterium* (C58C1)	pAgroLucR	4	97%	16%	13%
		52	65%	13%	11%
*Agrobacterium* (C58C1)	pAgroLucL	31	93%	23%	20%
		12	49%	13%	10%

^a^Luc activity > 3-fold that of the untransformed control

### Transgene rearrangements

Next, we tested the maintenance of the *Luc* transgene in the *Paro*-positive transformants obtained either through electroporation or through co-cultivation with *Agrobacterium*. The results (Additional file 2: Figure S2 shows the analysis of a subset of transformants) show that, although most transformants tested are PCR-positive for the *Paro* gene, only few carry the *Luc* gene. The frequency of *Luc*-positive colonies was tested in two independent experiments and compared to that obtained in electroporation experiments using the same vector. The results (Table 2) indicate that only 13%–16% of Paro-resistant colonies obtained through *Agrobacterium* transformation carry a complete *Luc* gene, compared to 26%–33% obtained through electroporation with the same vector. Thus, in contrast with the results obtained in higher plants [12], *Agrobacterium*-mediated transformation of *Chlamydomonas* does not alleviate, compared to electroporation, the rearrangement of transgenes cloned within the T-DNA borders.

**Fig. 2:**
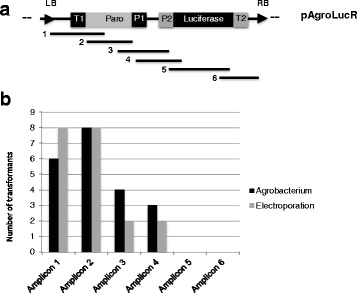
Deletions along the T-DNA in transformants obtained with electroporation or *Agrobacterium*-mediated transformation. *Chlamydomonas* cw15 cells were transformed with the pAgroLucR plasmid, using *Agrobacterium* or electroporation. Integrity of the inserted T-DNA was tested by PCR amplification with nested pairs of oligonucleotides spanning the T-DNA (Panel **a**) in a set of nine independent transformants. The histogram (Panel **b**) shows that there is a gradient of deletion from the LB (Amplicon1) to the RB (Amplicon 6) independently of the transformation method used (*Agrobacterium,* *black*; electroporation, *gray*). Oligonucleotide sequences are reported in Additional file 7: Table S2

PCR reactions on a set of nine independent transformants (Fig. 2, Additional file 3: Figure S3 and Additional file 4: Figure S4) with nested pairs of oligonucleotides spanning the whole T-DNA region (Fig. 2a) indicated that, both in *Agrobacterium*-mediated transformation and in electroporation, deletion frequency is highest close to the right border (RB), i.e. away from the *Paro* selectable marker (Fig. 2b).

### Influence of orientation with respect to the T-DNA borders on transgene rearrangements

**Fig. 3:**
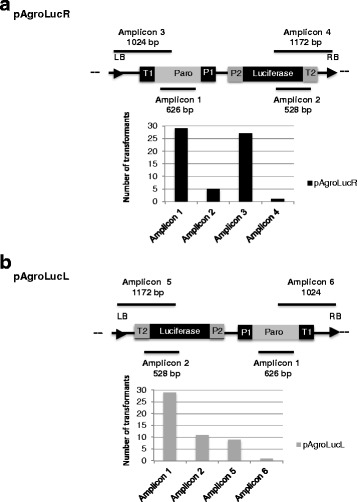
T-DNA deletion pattern in pAgroLucR and pAgroLucL transformants. The pAgroLucR and pAgroLucL plasmids were used for *Agrobacterium* mediated transformation and integrity of the T-DNA in the resulting transformants was tested via PCR with appropriate oligonucleotide pairs. Panels **a** (pAgroLucR) and **b** (pAgroLucL) show the position of the amplicons (top in each box) and the frequency of transformants showing amplification for each amplicon (bottom in each box). A set of 29 independent transformants were tested for each construct. All transformants contain amplicon 1, corresponding to the *Paro* gene. When *Luc* is cloned next to the LB is retained at higher frequency (pAgroLucL, amplicons 2 and 5 in Panel **b**) with respect to the RB (pAgroLucR, amplicons 2 and 4). See also Additional file 5: Figure S5 and Additional file 6: Figure S6. Oligonucleotide sequences are reported in Additional file 7: Table S3

T-DNA integration occurs in a directional way, resulting in asymmetric deletion frequencies in regions close to the right and left borders (RB and LB) [20]. In order to investigate if the position of the *Luc* gene with respect to LB and RB influences the frequency of deletions at this locus, we constructed a second vector, pAgroLucL, in which the orientation of the *Paro* and *Luc* genes is inverted with respect to pAgroLucR (Fig. 1c). The results (Table 2) indicate a slight increase in the number of the transformants containing an intact *Luc* transgene when the pAgroLucL construct was used. Deletions coming from the RB and LB as well as more internal deletions were tested via PCR (Fig. 3, Additional file 5: Figure S5 and Additional file 6: Figure S6). The results show that deletions of the *Luc* transgene are more frequent (82% vs 62%) when it flanks the RB compared to the LB.

### Transgene expression and its stability over time under selective pressure

Next, we asked whether transgene rearrangement or transgene silencing were the major causes resulting in loss of transgene expression. In order to answer this question, cells containing an intact *Luc* gene as judged by PCR were subjected to 3 subsequent subcultures on selective medium in order to get rid of the *Agrobacterium* cells and then assayed for Luc activity. The results (Table 2 and Additional file 7: Table S4) indicate that a large fraction of transformants containing an intact *Luc* transgene also showed some level of Luc activity (at least three times higher than the background) and that gene silencing may be slightly more active during electroporation than during *Agrobacterium*-mediated transformation.

**Fig. 4:**
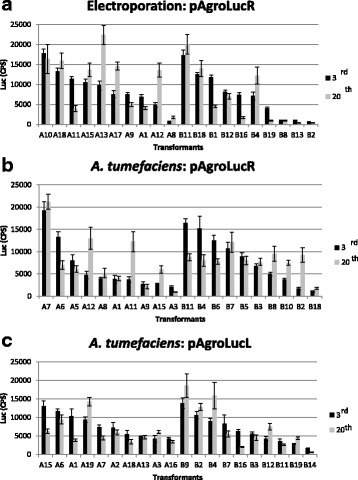
Temporal stability of Luc activity in *Chlamydomonas* transformants obtained through electroporation or *Agrobacterium*-mediated transformation. Twenty *Chlamydomonas* transformants showing the highest level of luciferase expression were collected from two experiments (experiments A and B: ten transformants chosen for each experiment, see Additional file 7: Table S4) and analyzed for luciferase expression at the 3rd (*black bars*) and the 20th (*gray bars*) cycles of subculture after the initial transformation event. **a** Electroporation with pAgroLucR **b** *Agrobacterium-*mediated trasformation with pAgroLucR **c** *Agrobacterium-*mediated trasformation with pAgroLucL. Luminescence is expressed as CPS normalized for 10^5^ cells

In order to follow the stability of *Luc* expression in transformants grown under selective pressure, cells were subcultured on solid medium containing paromomycin at 1-week intervals, and Luc activity was measured at the 3rd and 20th subculture cycle (Additional file 7: Table S4). In all three cases (electroporation and co-cultivation with *Agrobacterium* carrying pAgroLucR and pAgroLucL vectors), the percentage of *Luc*-positive transformants showing luciferase activity does not drop significantly from the 3rd to the 20th subculture cycle. Quantitative Luc activity data on the twenty highest expressors (Fig. 4) show that enzymatic activity is quite stable over time, indicating that neither the method of transformation (electroporation or *Agrobacterium*) nor the vector (pAgroLucL or pAgroLucR) influences Luc expression levels and their stability over subsequent generations on selective medium.

### Whole genome sequencing of electroporation- vs *Agrobacterium*-generated transformants

**Fig. 5:**
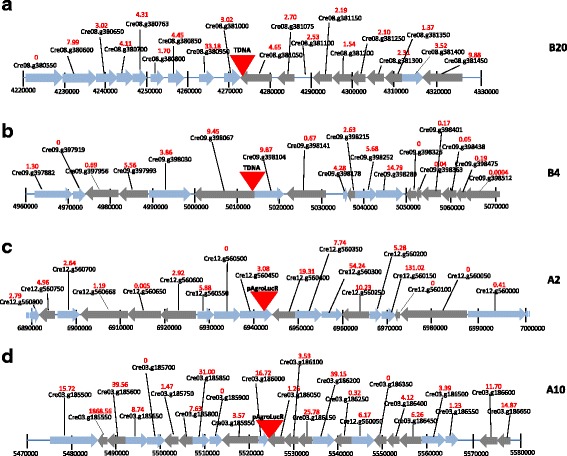
Examples of DNA insertion in transgenic *C. reinhardtii* clones. **a** and **b** Low and high Luc expressor transformants obtained by *Agrobacterium*-mediated transformation. **c** and **d** Low and high Luc expressor transformants obtained by electroporation. Insertions are shown as *red triangles*. Transformant names are shown on the *right*. Genes are indicated by *arrows* oriented according to their transcription direction and are labeled with gene IDs [32]. Gene expression (FPKM) in *C. reinhardtii* cells grown in TAP medium [32, 36] is reported in *red* above each gene ID

In order to identify the insertion points of transformants obtained with the different methods, we performed whole genomic sequencing of twenty transformants in the cw15 background, ten obtained by *Agrobacterium* and 10 by electroporation using the pAgroLucR plasmid. We selected 5 high and 5 low Luc expressors for each transformation method (*Agrobacterium* or electroporation) in order to assess any influence of the genomic context of the insertion on transgene expression. Paired-end Illumina reads (2 × 100 nucleotides long) were generated to give an average coverage of about 20×. The reads were aligned on the *Chlamydomonas* genome and the pAgroLucR vector sequences and the insertion points where determined using paired ends mapping to both the algal genome and the vector. The results are summarized in Table 3. One of the *Agrobacterium* transformants did not carry a detectable insertion, presumably because the insertion occurred in a genomic region not present in the available genome assembly. The remainder of the insertions occurred, for the most part, in genes (5′ and 3′ UTR and exons) with only a minority occurring in intergenic regions. One *Agrobacterium* and three electroporation transformants carried insertions in multiple chromosomal regions. Examples of DNA integration are given in Fig. 5 for one low and one high Luc expressor obtained by both *Agrobacterium*-mediated transformation and electroporation.

**Table 3:** Information on the insertion sites and Luc expression of the different transformants. Five low and five high Luc expressors were collected and analyzed for the characterization of the insertion site both for *Agrobacterium* transformation and electroporation. The first column reports the name of the transformant selected (see Additional file 7: Table S4). Luc activity of the transformants at the 3rd and 20th subculture in selective medium is expressed as average values of CPS (counts per second) normalized for 10^5^ cells ± standard deviation of three biological replicates. Chr #: chromosome number in which DNA integration occurred. Insertion points were determined from the *Chlamydomonas reinhardtii* structural annotation v5.5 [32]. Gene annotation was taken from the *Chlamydomonas reinhardtii* functional annotation v 5.5 [32]. Gene expression refers to RNA-Seq experiments conducted on wild type cells grown in TAP medium [32, 36] and are reported in FPKM (fragments per kilobase per million mapped fragments)

Transformant	Luc activity 3rd subculture	Luc activity 20th subculture	Chr #	Insertion point	Gene name	Gene annotation	Gene expression (FPKM)
	CPS ± sd	CPS ± sd					
Transformation method: *Agrobacterium*							
B 20	9 ± 2	8 ± 1	8	Gene (3′ UTR)	Cre08.g381050	Senescence-associated gene 12	4.65
A 14	38 ± 11	18 ± 2	1	Gene (exon)	Cre01.g051900	Ubiquinol-cytochrome C reductase iron-sulfur subunit	166.65
B 9	5 ± 1	64 ± 12	12	Gene (5′UTR)	Cre12.g486000	n.a.	1.49
B 12	7 ± 1	69 ± 13	17	Gene (exon)	Cre17.g736700	n.a.	0
A 18	48 ± 10	16 ± 2	1	Gene (exon)	Cre01.g015250	DNA binding; DNA-directed DNA polymerases	4.13
A 6	13,306 ± 1218	6979 ± 926	3	Gene (exon)	Cre03.g204200	n.a.	0
A 7	19,240 ± 1945	21,210 ± 1694	n.d.	n.d.	n.d.	n.d.	n.d.
B 4	15,202 ± 2795	8006 ± 1259	9	Gene (5′UTR)	Cre09.g398067	Rotamase FKBP 1	9.45
B 6	12,460 ± 1239	7751 ± 643	17	Gene (exon)	Cre17.g699600 Cre08.g377150	Sedoheptulose-bisphosphatase	14.62
			8	Gene (exon)		n.a.	18.19
B 11	16,458 ± 892	8762 ± 831	8	Gene (3′ UTR)	Cre08.g379400	n.a.	2.95
Transformation method: Electroporation							
A 2	7 ± 3	49 ± 6	12	Gene (3′ UTR)	Cre12.g560450	n.a.	3.08
A 3	2 ± 0	68 ± 9	1	Gene (exon)	Cre01.g014150	MATE efflux family protein	5.20
			4	Gene (exon)	Cre04.g223550	n.a.	0.42
A 4	4 ± 1	24 ± 5	13	Gene: 3′ UTR	Cre13.g569850	Ammonium transporter 1;2	0.89
A 5	37 ± 11	61 ± 7	12	Gene (exon)	Cre12.g559450	Calcium-dependent lipid-binding family protein	37.98
A 7	1 ± 1	21 ± 5	11	Gene (exon)	Cre11.g467556	n.a.	2.73
			5	Gene (5′UTR)	Cre05.g232100	n.a.	6.87
A 10	17,849 ± 1022	16,393 ± 3650	3	Gene (3′ UTR)	Cre03.g186050	n.a.	1.26
A 11	11,347 ± 645	3932 ± 771	2	2 Genes, both 3′UTR	Cre02.g098700;	ABC-2 type transporter family protein	0.63
				Intergenic region	Cre02.g098750	STELAR K+ outward rectifier	2.77
			8		/		/
B 11	17,253 ± 1316	20,099 ± 2464	3	Gene (intron)	Cre03.g165700	Pyruvate decarboxylase-2	87.52
A 18	13,348 ± 753	15,958 ± 1918	3	Intergenic region	/	/	/
B 18	12,482 ± 499	13,977 ± 1959	16	Intergenic region	/	/	/

*n.d.* not determined, *n.a.* not available

## Discussion

Although *Agrobacterium* mediated transformation in *Chlamydomonas* was first reported in 2004 [6]*,* up to now it has not been widely adopted. Previous publications [6–8], demonstrate the successful expression of exogenous genes and, in one case, stable integration in the nucleus. It has been proposed that, similar to plants, transformation of *Chlamydomonas* by *Agrobacterium* could provide stable integration and lower copy numbers, potentially leading to fewer problems with transgene co-suppression and instability. However, no data on the rearrangements/insertion points and copy number are available for *Agrobacterium*-mediated transformants and, most importantly, no comparative data are available with widely used methods, such as electroporation.

The experiments described here indicate that the cw15 strain is more susceptible to *Agrobacterium* transformation than the CC125 strain. This may be due to the fact that cw15 is a cell wall deficient mutant and may be more susceptible to *Agrobacterium* infection. Analysis of a large set of transformants indicated that, regardless of the method of transformation used, just a small percentage (10% to 20%) of the transformants show significant Luc expression (Table 2). In the case of electroporation, all the transformants analyzed contain the *Paro* selectable gene, while about 1/3 also contain a non-rearranged *Luc* gene. Transformation with *Agrobacterium* results in a higher number of false positive colonies (Table 2), lacking both genes. This may be due to low level expression of the *Paro* gene in *Agrobacterium*, leading to inactivation of paromomycin during the co-cultivation step, or to the long incubation of plates from *Agrobacterium*-mediated transformation, leading to light-mediated degradation of paromomycin, or to interference by the antibiotics used to remove *Agrobacterium* cells.

In cells transformed with pAgroLucR a gradient of deletions is observed, with very high deletion frequencies near the RB, i.e. away from the selectable marker gene (Fig. 2, Additional file 3: Figure S3 and Additional file 4: Figure S4). A slight effect of the LB and RB on deletion frequencies was observed, with the RB being more prone to deletion than LB, as observed in plants [20]. Luminometric assays performed at the 3th and 20th cycles of subculture in selective media after transformation revealed that the percentage of colonies expressing luciferase gene is relatively stable over time (Additional file 7: Table S4 and Fig. 4). This suggests that the major factors limiting *Luc* gene expression are the reporter gene rearrangements during the transformation step. We can not, however, exclude completely the occurrence of silencing phenomena in the first three subculture steps, since we analyzed Luc activity only after the third subculture in selective medium, to remove residual bacterial cells.

Sequencing of five high Luc and five low Luc expressors for each transformation method did not suggest any clear difference in insertion patterns or any correlation between Luc expression and the genomic region in which the insertion occurred (Table 3). A higher number of *Agrobacterium* insertions (10 out of 10) occurred in transcribed regions (exons and UTRs), compared to electroporation (9 out of 13). This is much higher than what reported in large scale insertional mutagenesis projects, in which only 50% of insertions were in exons and UTRs [21]. Also, *Agrobacterium* displayed a lower number of multiple insertions (1 out of 10 transformants) compared to electroporation (3 out of 10 transformants). This indicates that the optimized *Agrobacterium*-mediated transformation protocol reported here may be useful for insertional mutagenesis projects in *Chlamydomonas*.

Zhang et al. have proposed a model to explain the large rearrangements to which exogenous DNA is subjected during *Chlamydomonas* transformation. According to this model, the exogenous DNA is cleaved extracellularly by an endonuclease and then ligated to *Chlamydomonas* DNA present in the medium due to cell lysis, before it is taken up by the cell and inserted in the nuclear genome [21]. Our data only partially support this model. It is known that T-DNA is transferred from *Agrobacterium* to the recipient cell nucleus by means of a bacterial *pilus*, as a single-stranded complex with the virulence proteins VirD2 and VirE2, the former forming a covalent bond with the 5′ end of the T-DNA molecule [17]. During this process, it is unlikely that the T-DNA is exposed to extracellular endonucleases, and thus, if the cleavage was extracellular, we should observe a much lower frequency of DNA rearrangements in *Agrobacterium*-mediated transformants. Also if, as has been proposed, the mechanisms mediating transgene rearrangement in *Chlamydomonas* have an evolutionary significance as a defense against pathogenic bacteria and viruses, it makes more evolutionary sense that these mechanisms act within the *Chlamydomonas* cell, where the bacterial or viral DNA is naked, rather than in the extracellular space, where it is contained within a bacterial cell or a viral capsid. We thus propose that the introduction of exogenous DNA in the *Chlamydomonas* cell triggers an endonucleolytic mechanism aimed at its degradation, and that this mechanism preferably acts within the nucleus, when the nucleoprotein T-DNA complex is disassembled and the T-DNA is naked, before insertion into the nuclear DNA.

## Conclusions

*Chlamydomonas* has been proposed as a system for heterologous protein expression [22]. However, low levels of expression and rearrangements of the transforming DNA prevent it from being a competitive production platform with respect to bacterial, fungal or animal cells. In the present work we compared *Agrobacterium-*mediated transformation with electroporation of *Chlamydomonas* in order to verify if the former can alleviate these problems. Regardless of the method used to transform *Chlamydomonas* cells, extensive rearrangements occur at the *Luc* gene, which is not under selective pressure. This drawback was only partially compensated by the reduced *Luc* silencing in *Agrobacterium* transformants. Thus, differently from what observed in plants, *Agrobacterium*-mediated transformation does not present significant advantages in terms of higher or more stable expression. In order to increase transgene stability and expression, mutants affected in transgene silencing [23] or in exogenous DNA cleavage can be of great help. The knowledge gained from *Agrobacterium*-mediated transformation may be instrumental in the latter endeavour.

## Methods

### *Chlamydomonas* strains and culture conditions

The *Chlamydomonas reinhardtii* cell wall-deficient cw15 and cell wall-proficient CC125 (+) strains [24], were used for all experiments. Cells were grown photomixotrophically in Tris Acetate Phosphate (TAP) medium [24] at 25 °C under continuous irradiation with fluorescent white light (60 μE m^− 2^ s^− 1^). In the case of cw15 strain the growth medium was supplemented with 1% (*w*/*v*) D-sorbitol.

For luciferase assay, the transformants were grown in 1.5 ml of TAP medium at 160 rpm at 80 μE m^− 2^ s^−1^on a rotary shaker until they reach the stationary phase (about 48 hs) in 24-well blocks (Qiagen, cat. n. 19,583). The cultures were then diluted 1:20 in 4 ml of TAP medium and grown for 48 h. The plates were covered with Breathe-Easy membrane (Sigma-Aldrich, cat. n. Z763624-100EA) to prevent evaporation without limiting gaseous and light exchange. Frozen cell pellets relative to 200 μl of each culture were re-suspended in 40 μl of lysis buffer (Renilla Luciferase Assay System, Promega, cat. E2820), lysed at room temperature for 15 min on a rotary shaker (750 rpm) and then incubated on ice until assayed activity. Data were normalized using optical absorbance of at least two biological replicates for each transformant.

### Plasmid construction

In order to obtain the pAgroR (Fig. 1, panel a) and pAgroL plasmids (map not shown), pSL18 [25] was digested with NotI and KpnI. The digestion produces two fragments, 3255 and 2867 bp long, that were blunt ended using the Klenow Fragment (Roche, cat. n. 11,008,404,001). The 3255 bp fragment containing the paromomycin expression cassette and the *PSAD* promoter and terminator regulatory elements was gel purified and used for the next cloning step. The plasmid pKCRTI [26] (derived from pCAMBIA 1390) was digested with BglII and XhoI releasing four fragments of 13, 865, 3391 and 6906 bp long. The digestion was subjected to filling in by Klenow and the 6906 bp fragment containing all the genetic elements required for *Agrobacterium* propagation and transfer into the host nucleus (including the LB and RB) was gel purified. The two fragments of 3255 bp, originating from pSL18, and 6906 bp, originating from pKCRTI, were blunt cloned and PCR was performed using specific oligonucleotides to discriminate the two different orientations of the fragments. The suffix R and L in pAgro vectors refers to the position of the empty *PSAD* expression cassette respect to the borders (R: the *PSAD* cassette is close to the RB; L: the *PSAD* cassette is close to the LB). To build pAgroLucR and pAgroLucL plasmids, *Renilla reniformis* luciferase coding sequence was isolated by PCR from plasmid *PSAD*:cRLuc [27] with the following oligos: crLuc-EcoRI for CAGC**GAATTC***ATGGCCAGCAAGGTGTACGAC* and cRLuc-XbaI rev CAG**TCTAGA***TTACGTATCGTTCTTCAGC* (EcoRI and XbaI restriction sites are reported in bold, gene specific sequence in italics is underlined). The PCR product was then cloned EcoRI-XbaI into pAgroR and pAgroL generating respectively pAgroLucR and pAgroLucL (Fig. 1, panel b and c). The bacterial strain used for cloning was XL1Blue from Statagene. Bacterial transformation was carried out by electroporation.

### DNA extraction from *Chlamydomonas* cells and PCR analysis

Single colonies of *Chlamydomonas* cells were picked and resuspended in 200 μl of 5% (*w*/*v*) chelex solution (Sigma-Aldrich, cat. n. C7901-25G) in a 96-well plate. The plate was incubated 10 min at 100 °C, 10 min on ice and centrifuged at 6000 g for 3 min. One microliter of the supernatant was used to perform PCR analysis. The sequences of oligonucleotides used to screen *Chlamydomonas* transformants are reported in Additional file 7: Table S1. β-tubulin was used as positive control for DNA extraction, paromomycin oligonucleotides were used to screen the positivity to the antibiotic, kanamycin oligonucleotides were used to evaluate residual *Agrobacterium* contamination while cRLuc for 710 and *PSAD* Ter 276 were used to check the presence of luciferase gene.

*Chlamydomonas* colonies were analysed by PCR after the three transfers in 1,5% TAP agar plate containing 10 μg/μl paramomycin. In the case of *Chlamydomonas* transformants obtained through co-cultivation method 500 μg/μl cefotaxime and 500 μg/μl carbenicillin were added in order to remove residual *Agrobacterium* cells. Ninety-six colonies were analyzed by PCR for each transformation experiment.

### *Chlamydomonas* electroporation

*Chlamydomonas* cells were cultured in TAP medium to 3–6 × 10^6^ cells/ml and then centrifuged at 3000 g for 5 min at 4 °C. The pellet was washed once with cold TAP containing 60 mM D-sorbitol and resuspended at final concentration of 2 × 10^8^ cells/ml. 250 μl (5 × 10^7^ cells) of this culture was mixed with 200 ng of linearized vector in a 0,4 cm cuvette (BIORAD, cat. n. 165–2081) and electroporated at 25 μF, 2000 V/cm. The time constant ranged between 13 and 16 ms. The electroporated cells were recovered overnight in TAP medium at low light (30 μE m^− 2^ s^− 1^) and then plated on 1% TAP agar plate containing 10 μg/μl paramomycin in the case of vectors described in Fig. 1. For electroporation, the pAgroLucR vector (Fig. 1b) was linearized with AgeI. Two independent electroporation experiments were performed, for each electroporation ten transformations were carried out. Ninety-six transformants for each transformation were analysed by PCR.

### *Agrobacterium-*mediated transformation with published methods

In the first set of experiments, *Chlamydomonas* cells were transformed according to [28]. Briefly, LBA4404 (Takara cat. n° 9115) and C58C1 *Agrobacterium* cells, kindly donated by Prof. Edgardo Filippone, were transformed through electroporation with the pAgroR vector (Fig. 1, Panel a) and plated on LB containing 100 μg/ml kanamycin and 100 μg/ml rifampicin (LBA4404 strain) or 200 μg/ml kanamycin and 100 μg/ml rifampicin (C58C1 strain) and grown for 48 h at 28 °C. Five single colonies from each transformation plate were inoculated in 1.5 ml of LB medium containing the appropriate antibiotics and analysed by PCR in order to check the presence of the right and left borders with the following pairs of oligonucleotides: *PSAD* Ter for GATTTCGCTGATTGATACGG and R border rev TAAACGCTCTTTTCTCTTAGG, producing an amplicon of 894 bp, L border for TGGCAGGATATATTGTGGTG and Paro box rev CTGGACTGGGAGCGGTGT, producing an amplicon of 1039 bp. All the five picked colonies showed the right amplicons and one of them was inoculated in LB medium plus antibiotics to prepare glycerol stocks. For each transformation experiment 100 μl of glycerol stock were inoculated in 15 ml liquid YEB medium - containing 30 μg/ml rifampicin and 100 μg/ml kanamycin - and grown for 24 h at 28 °C. The cells where then centrifuged at 2700 g for 30 min and resuspended in liquid TAP medium containing 100 μM acetosyringone to an A_595_ of 0.5. 10^7^ *Chlamydomonas* cells were plated on a 90 mm petri dish and grown for two days at 80 μE m^− 2^ s^− 1^ in continuous light to allow a lawn of cells to be formed. 200 μl of the bacterial suspension obtained as described above was spread on the lawn of *Chlamydomonas* cells for co-cultivation. After co-cultivation for 48 h at 30 μE m^− 2^ s^− 1^ cells were harvested at 1500 g for 5 min washed twice with liquid TAP medium containing 500 μg/μl cefotaxime by centrifugation at 1500 g for 5 min. Finally the cells were resuspended in TAP at 5 × 10^7^ cells/ml concentration and 1 ml was then plated onto 1% agar TAP plates containing 10 μg/μl paramomycin, 500 μg/μl cefotaxime and 500 μg/μl carbenicillin. Paromomycin is the selective antibiotic for transformation while cefotaxime and carbenicillin were used to kill *Agrobacterium* cells. Single colonies appeared after 7 days of growth at 30 μE m^− 2^ s^− 1^. These colonies were picked and transferred on new TAP agar plates containing all the three antibiotics. Even if we could observe several colonies on the primary transformation plate, after two or three transfers on selective medium they died, probably because they were false positive or because the vector did not integrate stably into the nucleus. In a second round of experiments we followed the protocol described in [7], but in this case we could not observe any colony on the primary transformation plates.

### Development of a modified protocol for *Agrobacterium-*mediated transformation

In order to set up a protocol for *Chlamydomonas* transformation through *Agrobacterium* in our laboratory, we followed the indications by [19]. Glycerol stocks of the *Agrobacterium* strains LBA4404 and C58C1 transformed with pAgroR vector obtained as described in the previous paragraph were inoculated in 15 ml of AB medium (pH 7) [29] containing 0.5% (*w*/*v*) glucose, 30 μg/ml rifampicin and 100 μg/ml kanamycin. The flasks were grown at 28 °C for 24 h, then centrifuged at 2700 g for 30 min and finally resuspended in 40 ml of AB induction medium (AB medium containing 0.5% glucose, 20 μM MES pH 5,6 and 100 μM acetosyringone) and grown overnight at 28 °C. Before co-cultivation the cell density was adjusted to A_595_ = 0,5 with AB induction medium. Fifty ml of *Chlamydomonas* culture grown for 2 days in TAP medium at approximately 10^7^ cells/ml were centrifuged at 1500 g for 5 min and resuspended in AB medium at a final cell density of 10^8^ cells/ml. For co-cultivation, 100 μl of microalgae corresponding to 10^7^ cells and 40 μl of *Agrobacterium* were spotted on 1% agar AB induction medium plates. In total 14 spots, corresponding to 10^8^ *Chlamydomonas* cells, were plated on each 1% agar AB induction medium plate and the plates were grown at 30 μE m^− 2^ s^− 1^ for 48 h. The cells were then harvested from each plate, washed twice with liquid TAP medium containing 500 μg/μl cefotaxime, centrifuged at 1500 g for 5 min, resuspended in liquid TAP medium at a density of 5 × 10^7^ cells/ml and plated on two TAP agar plates containing 10 μg/μl paramomycin, 500 μg/μl cefotaxime and 500 μg/μl carbenicillin. After 1 week of growth, colonies started to appear. Transformation efficiency is expressed as an average of the number of paromomycin resistant colonies obtained per 10^8^ of *Chlamydomonas* cells.

Paromomycin resistant colonies were transferred for three subculture rounds on 1.5% TAP agar plates containing the selective antibiotics and then analyzed by PCR. Two independent transformation experiments for each vector were performed and 96 colonies for each experiment were analysed by PCR.

### Luciferase assay

Luciferase assay was performed using the Renilla Luciferase Assay System (Promega, cat. n. E2820) as described in [27]. The luminescence of the wild type (background luminescence) was subtracted from the luminescence of the transformants. All the transformants showing a level of Luc activity > 3 fold than the background were considered Luc-positive. The luminescence values reported are relative to 10^5^ cells. At least two biological and two technical replicates were analyzed for each transformant.

### Whole genome sequencing and bioinformatic analyses

Total DNA was extracted using the DNeasy Plant Mini Kit (Qiagen, Cat No. 69104) from pellets obtained from 8 ml of mid-log phase cultures (concentration of 5 × 10^6^ cells/ml), grown in TAP medium as described above.

DNA quality and concentration was checked by running samples on 0.5% agarose gel and by fluorometry (Qubit, ThermoFisher Scientific), respectively. For each sample, one microgram of DNA was used to generate paired-end Illumina libraries, which were then sequenced on a HiSeq 2500 Illumina sequencer generating about 8 millions paired-end reads on average. Reads were cleaned of adapters and quality trimmed with Cutadapt v. 1.8.1 [30] and Trimmomatic v. 0.33 [31]. A composite reference was prepared by combining *Chlamydomonas reinhardtii* genome v. 5.5 [32] and the pAgroLucR vector sequence. All libraries were aligned to the reference using Bowtie2 v. 2.2.7 [33] as single ends, to avoid any bias due to pairing in the alignment results (Additional file 7: Table S5). In order to isolate the insertion signals, only pairs with a hit both on the alga genome and the vector were retained. Finally, for each transformed sample, all signals were compared to the wild type and insertion points were called with MACS v. 2.1.1 [34].

## Additional files

Additional file 1: **Figure S1.** PCR confirmation of the presence of the *Paro* selectable marker. Fourteen Paro resistant colonies (1–14) obtained by co-cultivation of the cw15 strain with *Agrobacterium* C58C1 cells harbouring the pAgroR plasmid were analyzed by PCR for the presence of the endogenous *β-tub* gene (used as positive control for DNA extraction), *Paro* gene (selectable marker) and *Kan* gene (diagnostic of residual *Agrobacterium* contamination). Transformants 1–12 and 14 have the *Paro* gene, while the results on transformant 13 are inconclusive since the colony is still contaminated by *Agrobacterium*. Wt: cw15 strain; P: pAgroR plasmid; C+: positive cw15 transformant; C-: no DNA. M: 1 Kb Plus ladder (Life Technologies). Oligonucleotide sequences are shown in Additional file 7: Table S1. (PPTX 380 kb)

Additional file 2: **Figure S2.** Retention of the Luc transgene in a set of *Chlamydomonas* colonies transformed with the pAgroLucR plasmid. cw15 cells were co-cultivated with the *Agrobacterium* C58C1 strain harboring the pAgroLucR plasmid. The DNA extracted was analyzed for the presence of the genes shown in Additional file 1: Figure S1 plus the *Luc* gene. The vast majority of the transformants, although positive for the presence of the *Paro* gene, do not contain an intact *Luc* gene. A and B are two independent control transformants, positive for the presence of the *Paro* and *Luc* genes. P: pAgroLucR plasmid. C-: negative control. M: 1 Kb Plus DNA Ladder (Life Technologies). Oligonucleotide sequences are shown in Additional file 7: Table S1. (PPTX 1875 kb)

Additional file 3: **Figure S3.** PCR analysis on a set of nine independent transformants obtained through *Agrobacterium* to study the deletion pattern long the T-DNA. *Chlamydomonas* cw15 cells were co-cultivated with *Agrobacterium* C58C1 strain carrying the pAgroLucR plasmid. Six PCR reactions were performed on extracted DNA with nested pairs of oligonucleotides annealing in the T-DNA from the LB to the RB (Panel A). The results (Panel B) show that there is a gradient of deletions from the LB to the RB. β-tubulin was used as positive control for DNA extraction. M: 1 Kb Plus DNA Ladder (Life Technologies); wt: cw15 strain; P: pAgroLucR; −: negative control. Oligonucleotide sequences are reported in Additional file 7: Table S2. (PPTX 204 kb)

Additional file 4: **Figure S4.** PCR analysis on a set of nine independent transformants obtained through electroporation to study the deletion pattern along the T-DNA. *Chlamydomonas* cw15 cells were electroporated with the pAgroLucR plasmid. Six PCR reactions were performed on extracted DNA with nested pairs of oligonucleotides annealing in the T-DNA from the LB to the RB (Panel A). The results (Panel B) show that there is a gradient of deletions from the LB to the RB. β-tubulin was used as positive control for DNA extraction. M: 1 Kb Plus DNA Ladder (Life Technologies); wt: cw15 strain; P: pAgroLucR; −: negative control. Oligonucleotide sequences are reported in Additional file 7: Table S2. (PPTX 128 kb)

Additional file 5: **Figure S5.** Deletion pattern on the T-DNA in the pAgroLucR transformants obtained though co-cultivation of *Chlamydomonas* with *Agrobacterium* cells transformed with the pAgroLucR plasmid. The figure shows respectively a PCR analysis of a set of 29 independent cw15 transformants obtained with C58C1 *Agrobacterium* cells carrying the pAgroLucR vector. Wt: cw15, P: pAgroLucR plasmid; C-: negative control. Oligonucleotide sequences are reported in Additional file 7: Table S3. (PPTX 1196 kb)

Additional file 6: **Figure S6.** Deletion pattern on the T-DNA in the pAgroLucL transformats obtained though co-cultivation of *Chlamydomonas* with *Agrobacterium* cells transformed with the pAgroLucL plasmid. The Figure shows a PCR analysis of a set of 29 independent transformants obtained co-cultivating cw15 cells with C58C1 *Agrobacterium* cells carrying the pAgroLucL vector. Wt: cw15, P: pAgroLucL plasmid; C-: negative control. Oligonucleotide sequences are reported in Additional file 7: Table S3. (PPTX 2320 kb)

Additional file 7: **Table S1.** Oligonucleotides used to screen *Chlamydomonas* transformants. **Table S2.** Oligonucleotides used to study the T-DNA deletion pattern. **Table S3.** Oligonucleotides used to study the influence of R and L borders on T-DNA rearrangements. **Table S4.** Luciferase activity data at the 3rd and 20th subcultures. **Table S5.** NGS library mapping statistics. (DOCX 75 kb)

## Abbreviations

Chr: Chromosome
CPS: Counts per second
FPKM: Fragments per kilobase per million mapped fragments
*HSP70/RBCS2*: 70 kDa heat shock protein/ribulose bisphosphate carboxylase/oxygenase small subunit
LB: Left border
Luc: Luciferase
nt: Nucleotides
Paro: Paromomycin
*PSAD*: Photosystem I reaction center subunit II
RB: Right border
TAP: Tris Acetate Phosphate
UTR: Untraslated region
β-tub: β-tubulin
