# Supplementary figures and images for: Agrobacterium-mediated and electroporation-mediated transformation of Chlamydomonas reinhardtii: a comparative study
Source: BMC Biotechnol. 2018 Feb 17;18:11. doi: 10.1186/s12896-018-0416-3 (PMC5816537; doi:10.1186/s12896-018-0416-3)

## Slide 1
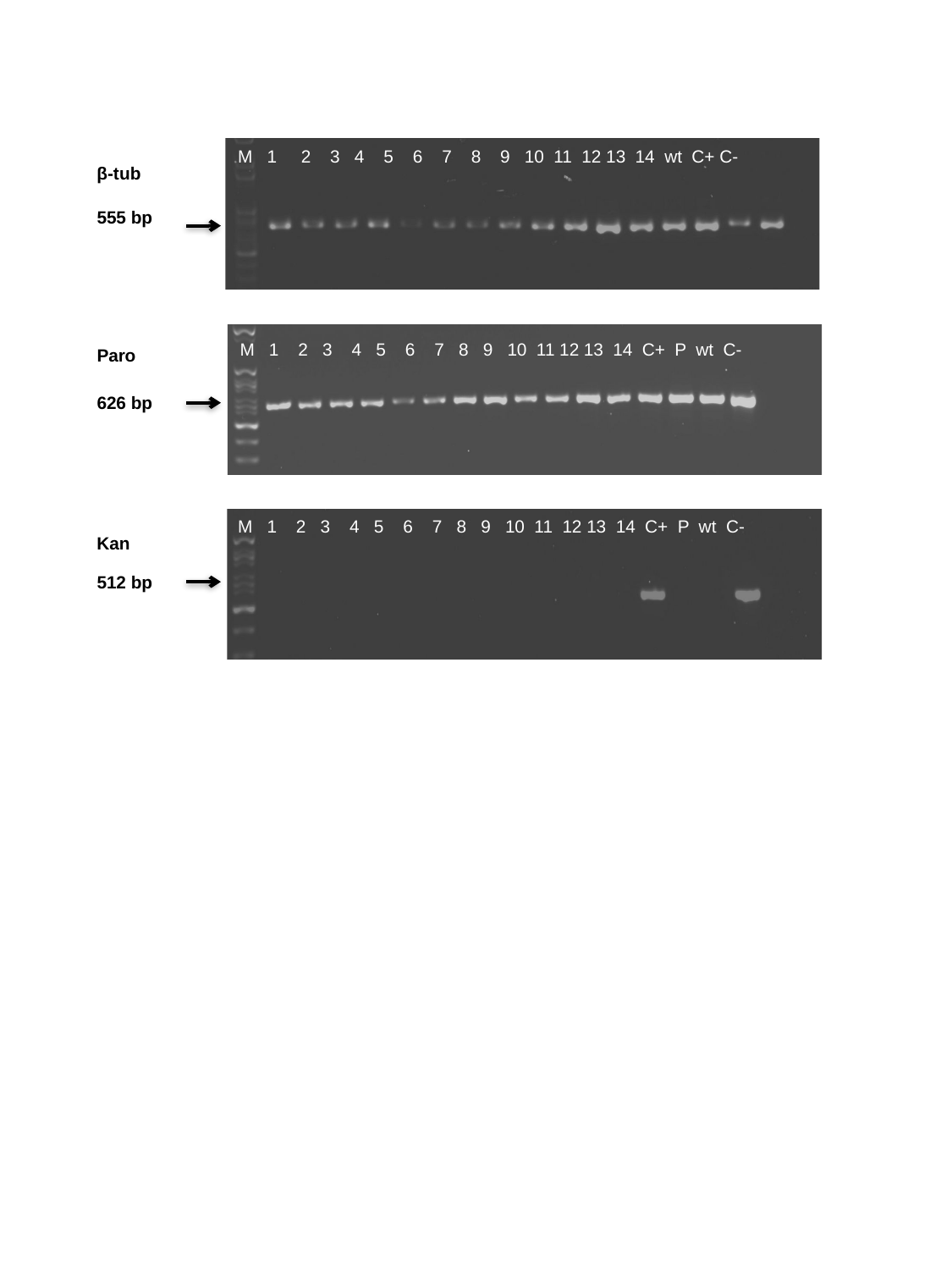

M 1 2 3 4 5 6 7 8 9 10 11 12 13 14 wt C+ C-
β-tub
555 bp
M 1 2 3 4 5 6 7 8 9 10 11 12 13 14 C+ P wt C-
Paro
626 bp
M 1 2 3 4 5 6 7 8 9 10 11 12 13 14 C+ P wt C-
Kan
512 bp

Supplement: Supplementary file 1 — Figure S1. PCR confirmation of the presence of the Paro selectable marker. Fourteen Paro resistant colonies (1–14) obtained by co-cultivation of the cw15 strain with Agrobacterium C58C1 cells harbouring the pAgroR plasmid were analyzed by PCR for the presence of the endogenous β-tub gene (used as positive control for DNA extraction), Paro gene (selectable marker) and Kan gene (diagnostic of residual Agrobacterium contamination). Transformants 1–12 and 14 have the Paro gene, while the results on transformant 13 are inconclusive since the colony is still contaminated by Agrobacterium. Wt: cw15 strain; P: pAgroR plasmid; C+: positive cw15 transformant; C-: no DNA. M: 1 Kb Plus ladder (Life Technologies). Oligonucleotide sequences are shown in Additional file 7: Table S1. (PPTX 380 kb) [file 12896_2018_416_MOESM1_ESM.pptx]

## Slide 1
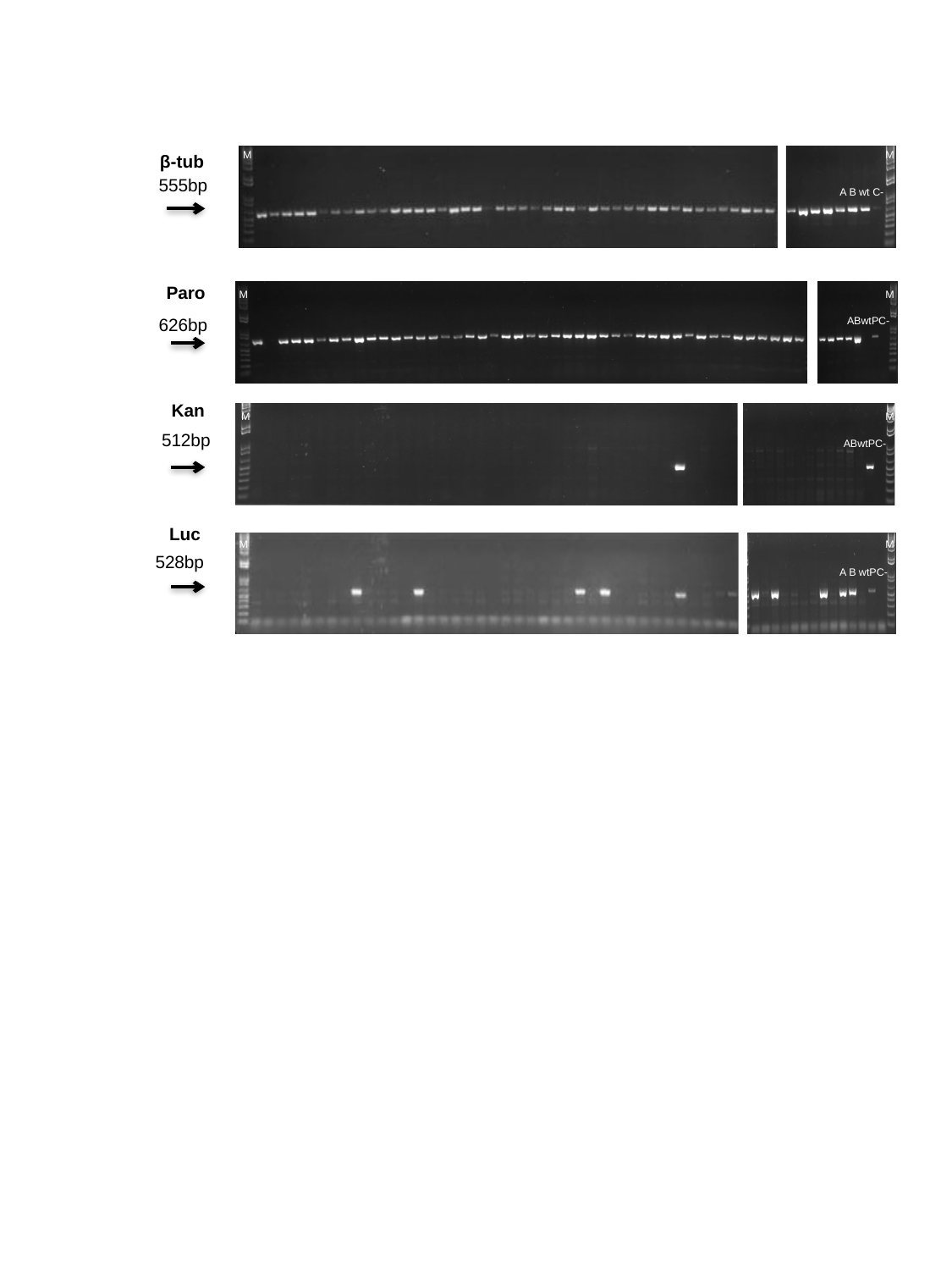

M
M
A B wt C-
β-tub
555bp
1 2 3 4 5 6 7 8 9 10 11 12 13 14 15 16 17 18 19 20 21 22 23 24 25 26 27 18 29 30 31 32 33 34 35 36 37 38 39 40 41 42 43 44
Beta
Paro
M
M
ABwtPC-
626bp
Kan
M
M
ABwtPC-
512bp
Luc
M
M
A B wtPC-
528bp

Supplement: Supplementary file 2 — Figure S2. Retention of the Luc transgene in a set of Chlamydomonas colonies transformed with the pAgroLucR plasmid. cw15 cells were co-cultivated with the Agrobacterium C58C1 strain harboring the pAgroLucR plasmid. The DNA extracted was analyzed for the presence of the genes shown in Additional file 1: Figure S1 plus the Luc gene. The vast majority of the transformants, although positive for the presence of the Paro gene, do not contain an intact Luc gene. A and B are two independent control transformants, positive for the presence of the Paro and Luc genes. P: pAgroLucR plasmid. C-: negative control. M: 1 Kb Plus DNA Ladder (Life Technologies). Oligonucleotide sequences are shown in Additional file 7: Table S1. (PPTX 1875 kb) [file 12896_2018_416_MOESM2_ESM.pptx]
