# Supplementary material for: Agrobacterium-mediated and electroporation-mediated transformation of Chlamydomonas reinhardtii: a comparative study
Source: BMC Biotechnol. 2018 Feb 17;18:11. doi: 10.1186/s12896-018-0416-3 (PMC5816537; doi:10.1186/s12896-018-0416-3)
Supplement: Supplementary file 4 — Figure S4. PCR analysis on a set of nine independent transformants obtained through electroporation to study the deletion pattern along the T-DNA. Chlamydomonas cw15 cells were electroporated with the pAgroLucR plasmid. Six PCR reactions were performed on extracted DNA with nested pairs of oligonucleotides annealing in the T-DNA from the LB to the RB (Panel A). The results (Panel B) show that there is a gradient of deletions from the LB to the RB. β-tubulin was used as positive control for DNA extraction. M: 1 Kb Plus DNA Ladder (Life Technologies); wt: cw15 strain; P: pAgroLucR; −: negative control. Oligonucleotide sequences are reported in Additional file 7: Table S2. (PPTX 128 kb) [file 12896_2018_416_MOESM4_ESM.pptx]

## Slide 1
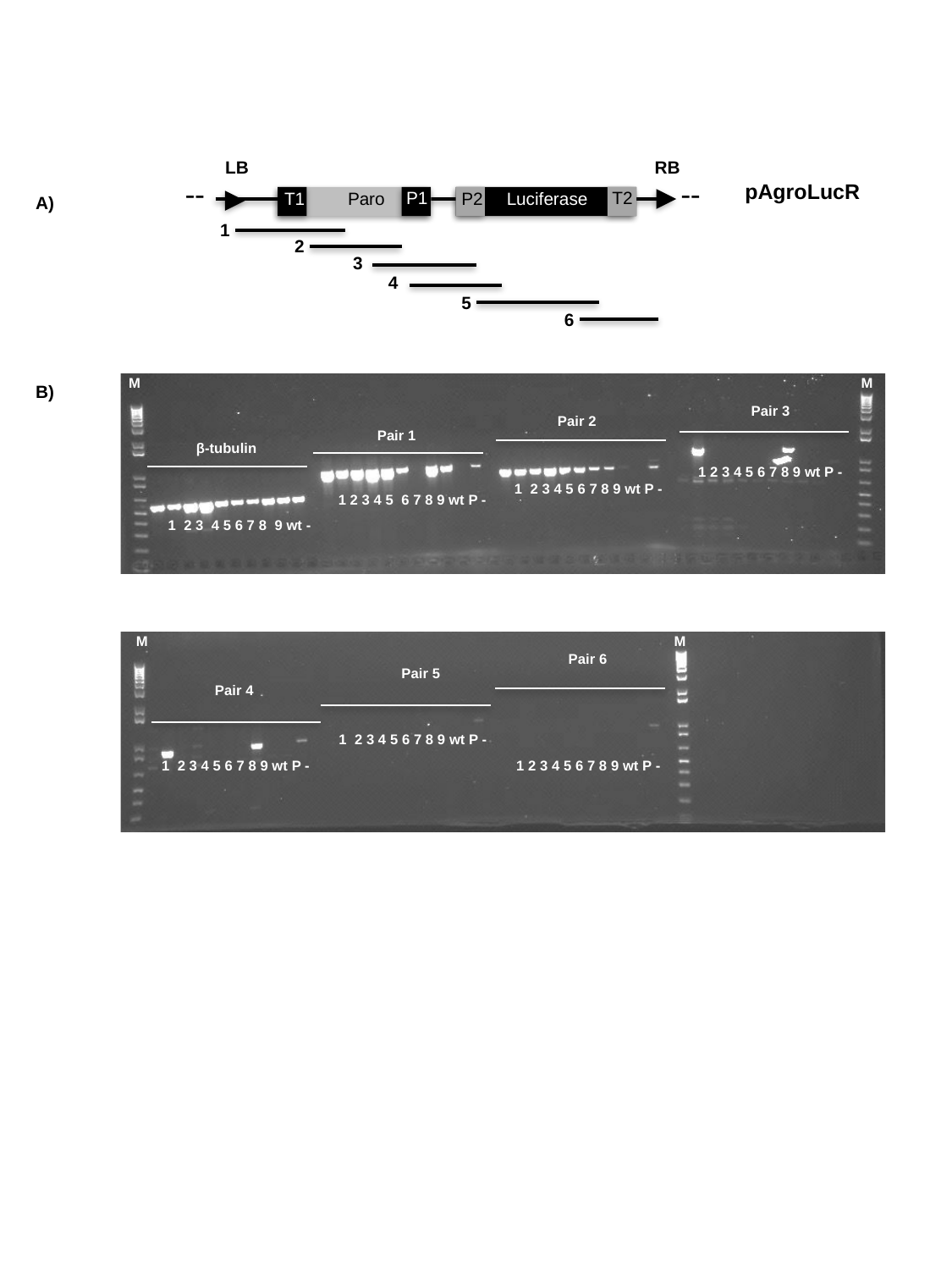

LB
RB
--
--
pAgroLucR
P1
T2
T1
Paro
P2
Luciferase
A)
1
2
3
4
5
6
M
M
B)
Pair 3
Pair 2
Pair 1
β-tubulin
1 2 3 4 5 6 7 8 9 wt P -
1 2 3 4 5 6 7 8 9 wt P -
1 2 3 4 5 6 7 8 9 wt P -
1 2 3 4 5 6 7 8 9 wt -
M
M
Pair 6
Pair 5
M
Pair 4
1 2 3 4 5 6 7 8 9 wt P -
1 2 3 4 5 6 7 8 9 wt P -
1 2 3 4 5 6 7 8 9 wt P -
