# Supplementary material for: Agrobacterium-mediated and electroporation-mediated transformation of Chlamydomonas reinhardtii: a comparative study
Source: BMC Biotechnol. 2018 Feb 17;18:11. doi: 10.1186/s12896-018-0416-3 (PMC5816537; doi:10.1186/s12896-018-0416-3)
Supplement: Supplementary file 5 — Figure S5. Deletion pattern on the T-DNA in the pAgroLucR transformants obtained though co-cultivation of Chlamydomonas with Agrobacterium cells transformed with the pAgroLucR plasmid. The figure shows respectively a PCR analysis of a set of 29 independent cw15 transformants obtained with C58C1 Agrobacterium cells carrying the pAgroLucR vector. Wt: cw15, P: pAgroLucR plasmid; C-: negative control. Oligonucleotide sequences are reported in Additional file 7: Table S3. (PPTX 1196 kb) [file 12896_2018_416_MOESM5_ESM.pptx]

## Slide 1
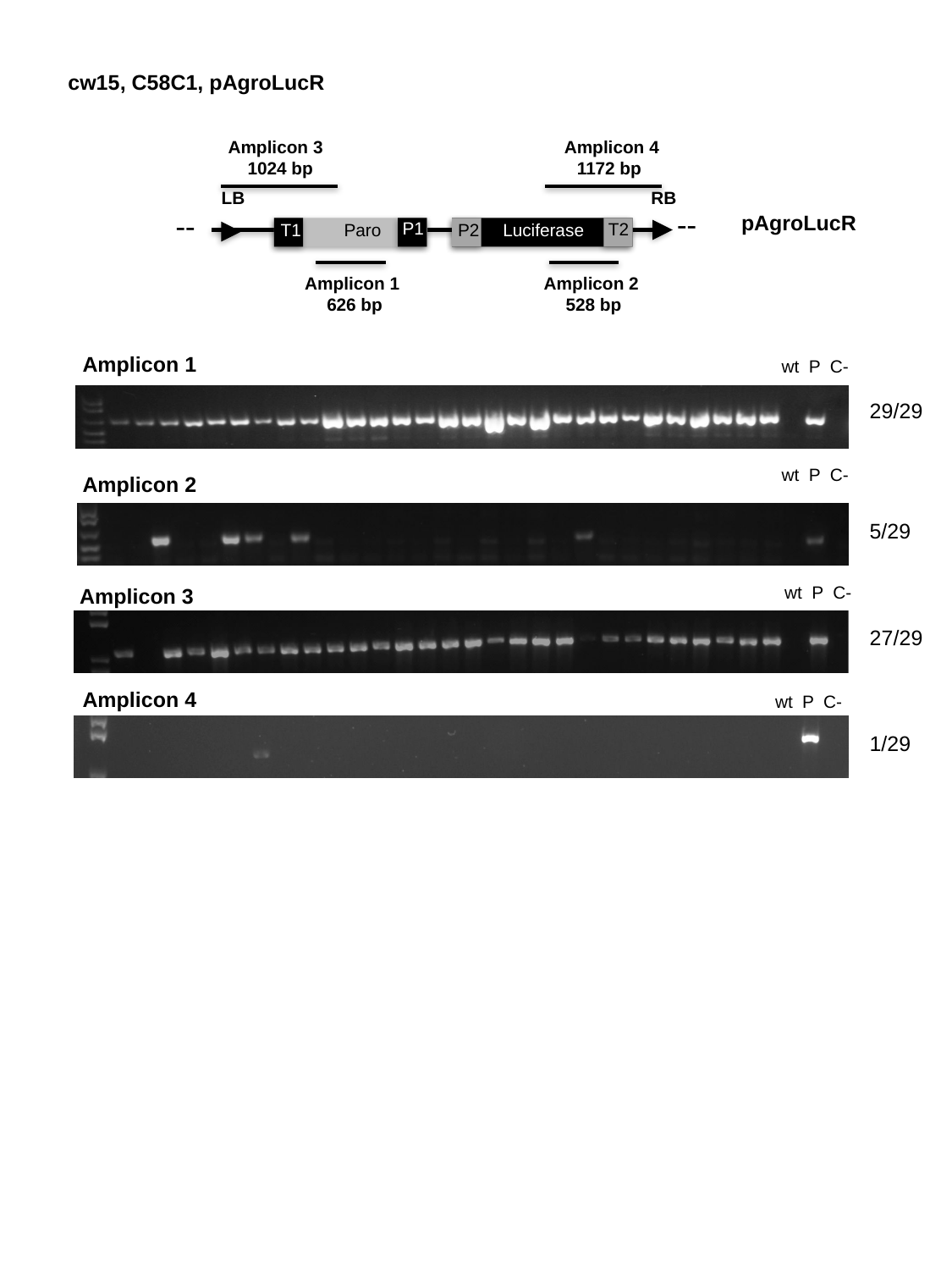

cw15, C58C1, pAgroLucR
Amplicon 3
 1024 bp
Amplicon 4
1172 bp
LB
RB
--
--
pAgroLucR
P1
T2
T1
Paro
P2
Luciferase
LB
Amplicon 1
626 bp
Amplicon 2
528 bp
Amplicon 1
wt P C-
29/29
wt P C-
Amplicon 2
5/29
wt P C-
Amplicon 3
27/29
Amplicon 4
wt P C-
1/29
