# Supplementary material for: Agrobacterium-mediated and electroporation-mediated transformation of Chlamydomonas reinhardtii: a comparative study
Source: BMC Biotechnol. 2018 Feb 17;18:11. doi: 10.1186/s12896-018-0416-3 (PMC5816537; doi:10.1186/s12896-018-0416-3)
Supplement: Supplementary file 6 — Figure S6. Deletion pattern on the T-DNA in the pAgroLucL transformats obtained though co-cultivation of Chlamydomonas with Agrobacterium cells transformed with the pAgroLucL plasmid. The Figure shows a PCR analysis of a set of 29 independent transformants obtained co-cultivating cw15 cells with C58C1 Agrobacterium cells carrying the pAgroLucL vector. Wt: cw15, P: pAgroLucL plasmid; C-: negative control. Oligonucleotide sequences are reported in Additional file 7: Table S3. (PPTX 2320 kb) [file 12896_2018_416_MOESM6_ESM.pptx]

## Slide 1
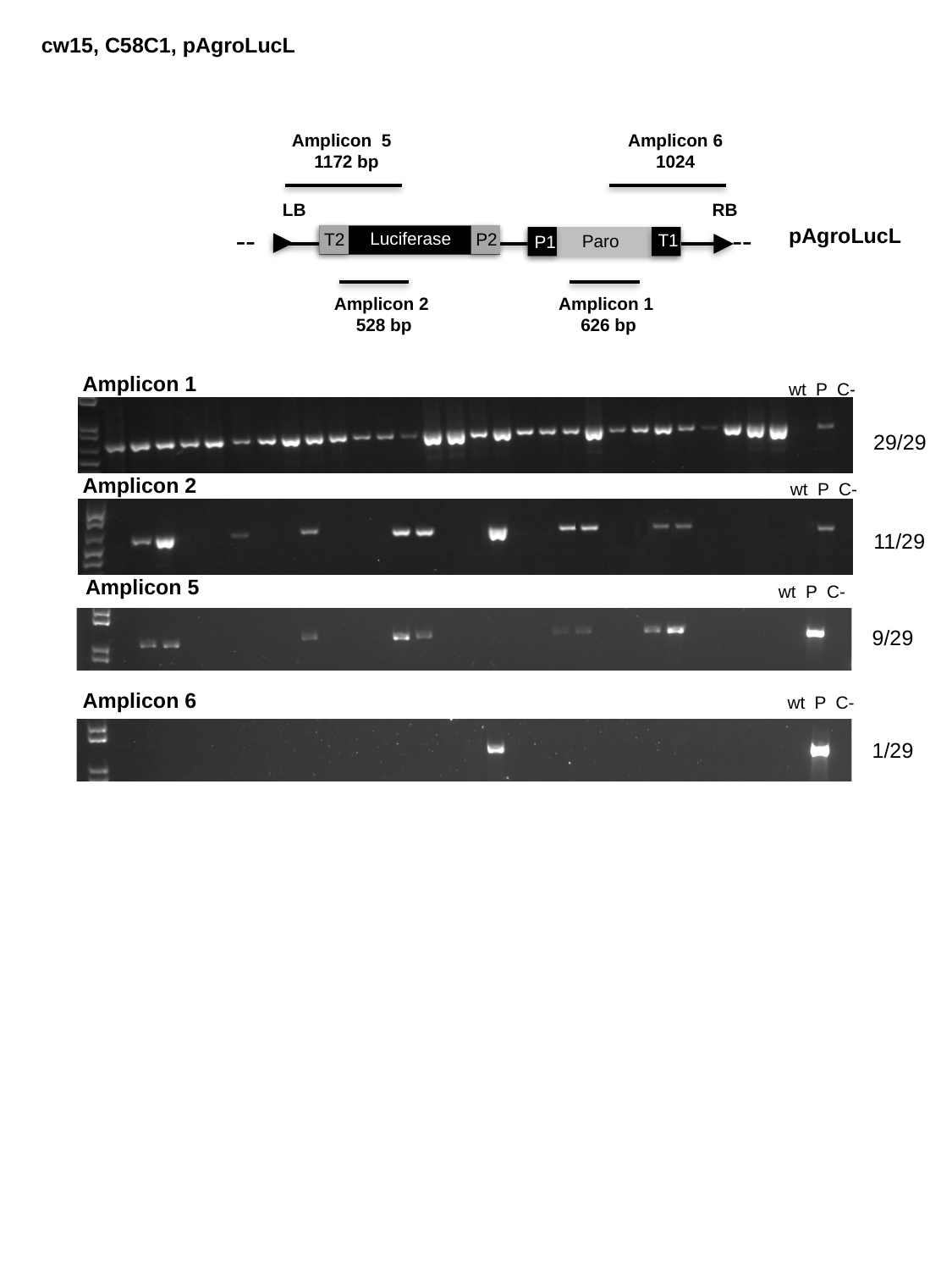

cw15, C58C1, pAgroLucL
Amplicon 5
 1172 bp
Amplicon 6
1024
LB
RB
--
--
pAgroLucL
Luciferase
T2
P2
T1
Paro
P1
Amplicon 2
528 bp
Amplicon 1
626 bp
Amplicon 1
wt P C-
29/29
Amplicon 2
wt P C-
11/29
Amplicon 5
1172bp
wt P C-
9/29
Amplicon 6
wt P C-
1/29
