# Supplementary material for: Agrobacterium-mediated and electroporation-mediated transformation of Chlamydomonas reinhardtii: a comparative study
Source: BMC Biotechnol. 2018 Feb 17;18:11. doi: 10.1186/s12896-018-0416-3 (PMC5816537; doi:10.1186/s12896-018-0416-3)
Supplement: Supplementary file 7 — Table S1. Oligonucleotides used to screen Chlamydomonas transformants. Table S2. Oligonucleotides used to study the T-DNA deletion pattern. Table S3. Oligonucleotides used to study the influence of R and L borders on T-DNA rearrangements. Table S4. Luciferase activity data at the 3rd and 20th subcultures. Table S5. NGS library mapping statistics. (DOCX 75 kb) [file 12896_2018_416_MOESM7_ESM.docx]

# Additional Tables

**Table S1 Oligonucleotides used to screen *Chlamydomonas* transformants.** β-tubulin was used as positive control for DNA extraction; the presence of paromomycin cassette was evaluated using oligonucleotides annealing to the *aphVIII* gene; presence of the luciferase gene was tested using cRLuc-for710 and PSAD-ter276 oligonucleotides in *Clamydomonas* cells transformed using pAgroLucL and pAgroLucR. In the case of *Agrobacterium*-mediated transformants, residual *Agrobacterium* contamination was checked using the Kan oligonucleotides.

| **Oligonucleotide name** | **Oligonucleotide sequence** | **Amplicon length** | **Target** |
| --- | --- | --- | --- |
| β-tub-for | TTGACTAAAGCAGGCGAGAC | 555 bp | β-tubulin |
| β-tub-rev | CTAAGCAGCCACGACGAAG |  |  |
| Paro-for | GCCAGCTTTTCCTCCGATAC | 626 bp | *aphVIII* gene |
| Paro-rev | GTGGGCTTGTTGGGTGAG |  |  |
| Kan-for | GGACGCAGAAGGCAATGTC | 512 bp | *Agrobacterium* kanamycin gene |
| Kan-rev | GATGTGGAACGGGAAAAGG |  |  |
| cRLuc-for710 | GCGCAACTACAACGCCTAC | 528 bp | Luciferase gene |
| PsaD-ter276 | TGGTCTTCTCAATGGGTGTG |  | *PsaD* terminator |

**Table S2 Oligonucleotides used to study the T-DNA deletion pattern.** PCR analysis shown in Figures 2 and in the Additional Figures 3 and 4 was performed using the oligonucleotides shown below.

| **Oligonucleotide name** | **Oligonucleotide sequence** | **Amplicon length** | **Amplicon number** |
| --- | --- | --- | --- |
| T-DNA 50 for | AACACATTGCGGACGTTTTT | 906 bp | 1 |
| T-DNA 936 rev | TCGACTTGGAGGATCTGGAC | 906 bp |  |
| T-DNA 699 for | GAACCACGGGTCCTCCTC | 805 bp | 2 |
| T-DNA 1484 rev | AGGCTTTTGGCTCCTCTGTC | 805 bp |  |
| T-DNA 1334 for | CCACAACAACCCACTCACAA | 1176 bp | 3 |
| T-DNA 2490 rev | CTCGGAACGACGACAAGAAG | 1176 bp |  |
| T-DNA 2019 for | ATAGCCCGCCAAATCAGTC | 887 bp | 4 |
| T-DNA 2886 rev | GTGAGTAGCAGTGGGGTCCT | 887 bp |  |
| T-DNA 2651 for | GCGGCGTGATTATTGGTATT | 1091 bp | 5 |
| T-DNA 3723 rev | GATGGCGTTGCTGAAGAAG | 1091 bp |  |
| T-DNA 3654 for | GCGCAACTACAACGCCTACT | 921 bp | 6 |
| T-DNA 4574 rev | CAAGACCGGCAACAGGATT | 921 bp |  |

**Table S3 Oligonucleotides used to study the influence of R and L borders on T-DNA rearrangements.** PCR analysis shown in Figure 3 and in Additional Figures 5 and 6 was performed using the oligonucleotides shown below.

| **Oligonucleotide name** | **Oligonucleotide sequence** | **Amplicon length** | **Amplicon number** |
| --- | --- | --- | --- |
| Paro for | GCCAGCTTTTCCTCCGATAC | 626 bp | 1 |
| Paro rev | GTGGGCTTGTTGGGTGAG |  |  |
| cRLuc for 710 | GCGCAACTACAACGCCTAC | 528 bp | 2 |
| PsaD Ter 276 | TGGTCTTCTCAATGGGTGTG |  |  |
| L border for 2 | TGTGGTGTAAACAAATTGACGC | 1024 | 3 |
| Paro box | CTGGACTGGGAGCGGTGT |  |  |
| cRLuc for 780 | GCGCAACTACAACGCCTAC | 1172 | 4 |
| R border REV 2 | TTTCTCTTAGGTTTACCCGCC |  |  |
| L border for 2 | TGTGGTGTAAACAAATTGACGC | 1172 bp | 5 |
| cRLuc for 780 | GCGCAACTACAACGCCTAC |  |  |
| R border REV 2 | TTTCTCTTAGGTTTACCCGCC | 1024 bp | 6 |
| Paro box | CTGGACTGGGAGCGGTGT |  |  |

**Table S4 Luciferase activity data at the 3^rd^ and 20^th^ subcultures.** Twenty transformants containing an intact *Luc* gene as judged by PCR were analyzed for each of two experiments (A and B). The luminescence value of the wild type was subtracted from the luminescence value of the transformants. For each transformant the CPS average and standard deviation of three biological replicates is reported. The transformants showing an average value of CPS higher than three times the background were considered positive to Luc assay.

| **Transformation method:** Electroporation | | | | |
| --- | --- | --- | --- | --- |
| **Plasmid:** pAgroLucR | | | | |
| **Experiment:** A | | | | |
| **Transformant** | **Luc activity 3^rd^ subculture** | | **Luc activity 20^th^subculture** | |
|  | **CPS** | **Sd** | **CPS** | **Sd** |
| A 1 | 6884 | 530 | 4123 | 384 |
| A 2 | 7 | 3 | 49 | 6 |
| A 3 | 2 | 0 | 68 | 9 |
| A 4 | 4 | 1 | 24 | 5 |
| A 5 | 37 | 11 | 61 | 7 |
| A 6 | 231 | 27 | 195 | 15 |
| A 7 | 1 | 1 | 21 | 5 |
| A 8 | 816 | 36 | 1755 | 303 |
| A 9 | 7509 | 402 | 4966 | 538 |
| A 10 | 17849 | 1022 | 16393 | 3650 |
| A 11 | 11347 | 645 | 3932 | 771 |
| A 12 | 4841 | 669 | 13570 | 1739 |
| A 13 | 9743 | 1147 | 22412 | 2359 |
| A 14 | 119 | 17 | 474 | 85 |
| A 15 | 10503 | 786 | 13640 | 1693 |
| A 16 | 9 | 3 | 32 | 14 |
| A 17 | 7582 | 824 | 14601 | 974 |
| A 18 | 13348 | 753 | 15958 | 1918 |
| A 19 | 24 | 3 | 69 | 26 |
| A 20 | 35 | 7 | 38 | 11 |
| **Experiment:** B | | | | |
| **Transformant** | **Luc activity 3^rd^ subculture** | | **Luc activity 20^th^ subculture** | |
|  | **CPS** | **Sd** | **CPS** | **Sd** |
| B 1 | 11757 | 548 | 4490 | 293 |
| B 2 | 520 | 118 | 345 | 41 |
| B 3 | 49 | 11 | 34 | 4 |
| B 4 | 7100 | 974 | 12201 | 2107 |
| B 5 | 18 | 6 | 7 | 4 |
| B 6 | 1 | 0 | 9 | 2 |
| B 7 | 6 | 1 | 11 | 2 |
| B 8 | 944 | 119 | 986 | 123 |
| B 9 | 2 | 1 | 5 | 2 |
| B 10 | 1 | 0 | 3 | 0 |
| B 11 | 17253 | 1316 | 20099 | 2464 |
| B 12 | 8182 | 428 | 7018 | 672 |
| B 13 | 845 | 233 | 348 | 66 |
| B 14 | 1 | 0 | 3 | 0 |
| B 15 | 54 | 5 | 38 | 4 |
| B 16 | 7415 | 610 | 1637 | 254 |
| B 17 | 13 | 2 | 14 | 3 |
| B 18 | 12482 | 499 | 13977 | 1959 |
| B 19 | 3957 | 327 | 913 | 143 |
| B 20 | 210 | 17 | 271 | 75 |
| **Transformation method:** *Agrobacterium* | | | | |
| **Plasmid:** pAgroLucR | | | | |
| **Experiment:** A | | | | |
| **Transformant** | **Luc activity 3^rd^ subculture** | | **Luc activity 20^th^ subculture** | |
|  | **CPS** | **Sd** | **CPS** | **Sd** |
| A 1 | 3789 | 940 | 3972 | 479 |
| A 2 | 1181 | 255 | 7696 | 1398 |
| A 3 | 2022 | 346 | 970 | 61 |
| A 4 | 907 | 61 | 1219 | 83 |
| A 5 | 7974 | 1342 | 6092 | 729 |
| A 6 | 13306 | 1218 | 6979 | 926 |
| A 7 | 19240 | 1945 | 21210 | 1694 |
| A 8 | 3897 | 344 | 5162 | 1055 |
| A 9 | 2746 | 481 | 2182 | 519 |
| A 10 | 122 | 12 | 32 | 3 |
| A 11 | 3740 | 615 | 12251 | 2226 |
| A 12 | 4747 | 880 | 12976 | 2486 |
| A 13 | 365 | 43 | 478 | 76 |
| A 14 | 38 | 11 | 18 | 2 |
| A 15 | 2728 | 196 | 5987 | 890 |
| A 16 | 234 | 47 | 326 | 39 |
| A 17 | 189 | 32 | 299 | 15 |
| A 18 | 48 | 10 | 16 | 2 |
| A 19 | 322 | 52 | 457 | 79 |
| A 20 | 194 | 25 | 279 | 69 |
| **Experiment:** B | | | | |
| **Transformant** | **Luc activity 3^rd^ subculture** | | **Luc activity 20^th^ subculture** | |
|  | **CPS** | **Sd** | **CPS** | **Sd** |
| B 1 | 376 | 26 | 2184 | 443 |
| B 2 | 1683 | 286 | 9251 | 1671 |
| B 3 | 6787 | 475 | 7735 | 781 |
| B 4 | 15202 | 2795 | 8006 | 1259 |
| B 5 | 8925 | 811 | 7990 | 979 |
| B 6 | 12460 | 1239 | 7751 | 643 |
| B 7 | 10665 | 1469 | 12134 | 2258 |
| B 8 | 4986 | 426 | 9499 | 1702 |
| B 9 | 5 | 1 | 64 | 12 |
| B 10 | 3744 | 262 | 7477 | 598 |
| B 11 | 16458 | 892 | 8762 | 831 |
| B 12 | 7 | 1 | 69 | 13 |
| B 13 | 345 | 189 | 1856 | 323 |
| B 14 | 215 | 49 | 316 | 29 |
| B 15 | 718 | 44 | 389 | 43 |
| B 16 | 480 | 298 | 206 | 138 |
| B 17 | 117 | 26 | 401 | 33 |
| B 18 | 994 | 178 | 1774 | 230 |
| B 19 | 305 | 87 | 585 | 57 |
| B 20 | 9 | 2 | 8 | 1 |
| **Transformation method:** *Agrobacterium* | | | | |
| **Plasmid:** pAgroLucL | | | | |
| **Experiment:** A | | | | |
| **Transformant** | **Luc activity 3^rd^ subculture** | | **Luc activity 20^th^ subculture** | |
|  | **CPS** | **Sd** | **CPS** | **Sd** |
| A 1 | 10350 | 1927 | 3868 | 319 |
| A 2 | 7144 | 1522 | 5901 | 689 |
| A 3 | 4251 | 902 | 6001 | 397 |
| A 4 | 3451 | 610 | 4138 | 718 |
| A 5 | 29 | 3 | 39 | 3 |
| A 6 | 11634 | 582 | 9429 | 1204 |
| A 7 | 7280 | 609 | 4481 | 520 |
| A 8 | 3839 | 323 | 4224 | 654 |
| A 9 | 3173 | 418 | 3712 | 329 |
| A 10 | 3758 | 461 | 1641 | 124 |
| A 11 | 3871 | 1157 | 38 | 7 |
| A 12 | 10 | 1 | 65 | 18 |
| A 13 | 4749 | 192 | 4634 | 469 |
| A 14 | 3235 | 175 | 3615 | 826 |
| A 15 | 12990 | 1471 | 6340 | 609 |
| A 16 | 4228 | 374 | 3380 | 320 |
| A 17 | 4111 | 504 | 6602 | 746 |
| A 18 | 5487 | 944 | 3454 | 566 |
| A 19 | 9407 | 780 | 14231 | 1128 |
| A 20 | 847 | 108 | 3422 | 571 |
| **Experiment:** B | | | | |
| **Transformant** | **Luc activity 3^rd^ subculture** | | **Luc activity 20^th^ subculture** | |
|  | **CPS** | **Sd** | **CPS** | **Sd** |
| B 1 | 14 | 3 | 63 | 12 |
| B 2 | 10601 | 1052 | 12821 | 964 |
| B 3 | 5571 | 552 | 4561 | 743 |
| B 4 | 8933 | 717 | 15865 | 3624 |
| B 5 | 14 | 4 | 21 | 5 |
| B 6 | 40 | 5 | 59 | 11 |
| B 7 | 8352 | 2332 | 5516 | 864 |
| B 8 | 705 | 79 | 442 | 65 |
| B 9 | 13841 | 1472 | 18607 | 3171 |
| B 10 | 24 | 2 | 36 | 4 |
| B 11 | 3503 | 645 | 2615 | 151 |
| B 12 | 4273 | 632 | 7440 | 890 |
| B 13 | 43 | 11 | 53 | 11 |
| B 14 | 1615 | 131 | 554 | 94 |
| B 15 | 188 | 37 | 403 | 99 |
| B 16 | 6290 | 458 | 2024 | 96 |
| B 17 | 12 | 1 | 56 | 6 |
| B 18 | 34 | 11 | 14 | 3 |
| B 19 | 2831 | 124 | 4390 | 379 |
| B 20 | 577 | 145 | 709 | 159 |

**Table S5 NGS library mapping statistics.**

Five low and five high Luc expressors containing the pAgroLucR plasmid were analyzed for each transformation method. The name of the transformants corresponds to those in Additional Table S4. The third column shows the number of paired-end reads with at least one alignment on the reference (*C. reinhardtii* genome and T-DNA). The fourth column shows the number of paired-end reads mapping to both the algal genome and the vector.

| **Transformation method** | **Transformant** | **Mapped pairs** | **Pairs mapping to the T-DNA-genome junction** | **Ratio** |
| --- | --- | --- | --- | --- |
| *Agrobacterium* | B 20 | 7,735,051 | 479 | 0.0062% |
| *Agrobacterium* | A 14 | 6,260,353 | 378 | 0.0060% |
| *Agrobacterium* | B 9 | 3,261,422 | 199 | 0.0061% |
| *Agrobacterium* | B 12 | 4,603,796 | 327 | 0.0071% |
| *Agrobacterium* | A 18 | 5,740,225 | 316 | 0.0055% |
| *Agrobacterium* | A 6 | 6,476,649 | 974 | 0.0150% |
| *Agrobacterium* | A 7 | 3,525,967 | 181 | 0.0051% |
| *Agrobacterium* | B 4 | 9,293,034 | 535 | 0.0058% |
| *Agrobacterium* | B 6 | 7,882,171 | 416 | 0.0053% |
| *Agrobacterium* | B 11 | 6,124,128 | 277 | 0.0045% |
| Electroporation | A 2 | 5,853,609 | 464 | 0.0079% |
| Electroporation | A 3 | 5,490,140 | 509 | 0.0093% |
| Electroporation | A 4 | 6,515,279 | 412 | 0.0063% |
| Electroporation | A 5 | 7,769,161 | 904 | 0.0116% |
| Electroporation | A 7 | 5,321,661 | 391 | 0.0073% |
| Electroporation | A 10 | 6,176,898 | 848 | 0.0137% |
| Electroporation | A 11 | 7,310,510 | 568 | 0.0078% |
| Electroporation | B 11 | 7,166,101 | 436 | 0.0061% |
| Electroporation | A 18 | 6,362,177 | 522 | 0.0082% |
| Electroporation | B 18 | 7,184,534 | 535 | 0.0074% |
| WT |  | 9,573,173 | 315 | 0.0033% |
